# Identification of an ACE-Inhibitory Peptide from Walnut Protein and Its Evaluation of the Inhibitory Mechanism

**DOI:** 10.3390/ijms19041156

**Published:** 2018-04-11

**Authors:** Cong Wang, Maolin Tu, Di Wu, Hui Chen, Cheng Chen, Zhenyu Wang, Lianzhou Jiang

**Affiliations:** 1 College of Food Science, Northeast Agricultural University, Harbin 150030, China; yuanque@163.com; 2 Department of Food Science and Engineering, Harbin Institute of Technology, Harbin 150090, China; tu000035@umn.edu; 3 School of Food Science and Technology, National Engineering Research Center of Seafood, Dalian Polytechnic University, Dalian 116034, China; m13039998695@163.com (D.W.); realcrital@126.com (H.C.); 6170206002@stu.jiangnan.edu.cn (C.C.); wangzhenyu@dlpu.edu.cn (Z.W.)

**Keywords:** walnut, protein, peptides, ACE inhibitory, molecular docking

## Abstract

In the present study, a novel angiotensin I-converting enzyme inhibitory (ACE inhibitory) peptide, EPNGLLLPQY, derived from walnut seed storage protein, fragment residues 80–89, was identified by ultra-high performance liquid chromatography electrospray ionization quadrupole time of flight mass spectrometry (UPLC-ESI-Q-TOF-MS/MS) from walnut protein hydrolysate. The IC_50_ value of the peptide was 233.178 μM, which was determined by the high performance liquid chromatography method by measuring the amount of hippuric acid (HA) generated from the ACE decomposition substrate (hippuryl-l-histidyl-l-leucine (HHL) to assess the ACE activity. Enzyme inhibitory kinetics of the peptide against ACE were also conducted, by which the inhibitory mechanism of ACE-inhibitory peptide was confirmed. Moreover, molecular docking was simulated by Discovery Studio 2017 R2 software to provide the potential mechanisms underlying the ACE-inhibitory activity of EPNGLLLPQY.

## 1. Introduction

There is a trend that the number of people with cardiovascular disease (CVD) is increasing year by year. More than 17.5 million people died due to CVD every year, which was reported by WHO [1], and CVD has been globally considered as the leading cause of death [2]. Hypertension is one of the remarkable risk factors for CVD with typical characteristics of age-related disorders [3,4,5]. Angiotensin I-converting enzyme (ACE, EC3.4.15.1) plays a vital role in blood pressure regulation in vivo through both the renin-angiotensin system (RAS) and the kallikrein-kinin system (KKS). In the RAS, the potent vasopressor angiotensin II is released by the cleavage of the C-terminal dipeptide from angiotensin I [6], which is induced by ACE. In the KKS, the vasodilator bradykinin is inactivated by ACE through the removal of the two C-terminal dipeptides [7]. Consequently, ACE-inhibitors (ACEIs) are used to lower blood pressure [8].

Many potent synthetic ACE inhibitors have been used for the clinical treatment of hypertension in the human body [9]. However, these synthetic ACE inhibitors have various side effects, such as hypotension, coughing, increasing potassium level and fetal abnormalities [10]. Therefore, the ACE inhibitors derived from food materials have attracted more attention [11,12]. Many ACE-inhibitory peptides have been identified from food-derived proteins, including bovine milk, plant proteins, such as soybean and wheat germ protein, and marine proteins [13,14,15,16].

Walnut can be used as an ingredient in other food stuffs. Walnut protein has been investigated as an excellent origin of food with antihypertensive properties. Some studies showed that the bioactive peptides from enzymatic hydrolysates of proteins showed better bioactivities than the proteins [17,18,19].

The relationship between the amino acid sequence and the bioactivity of peptides is a hot topic in this field, which is attracting more attention. In the previous studies, several ACE-inhibitory peptides from walnut proteins had been purified and identified [12,18,20]. Moreover, the molecular docking method can provide some simulation interaction information between the receptor and the biological macromolecular ligand based on the ligand and receptor lock and key principle [21], which can be used to analyze the interactions between the peptide and ACE [22].

In this study, the walnut protein hydrolysate was prepared by enzymatic digestion, and the sequence of the derived peptides was determined by UPLC-Q-TOF-MS/MS. A novel ACEI peptide was screened through the in silico method (Peptide Ranker and molecular docking) [23] and identified by UPLC-Q-TOF-MS/MS. The IC_50_ value of the target peptide was determined. Moreover, the ACE-inhibitory kinetics of the peptide and the potential mechanism of bioactivity were proposed by using molecular docking analysis.

## 2. Results and Discussion

### 2.1. Identification of Peptides from Walnut Protein Hydrolysates

**Figure 1:**
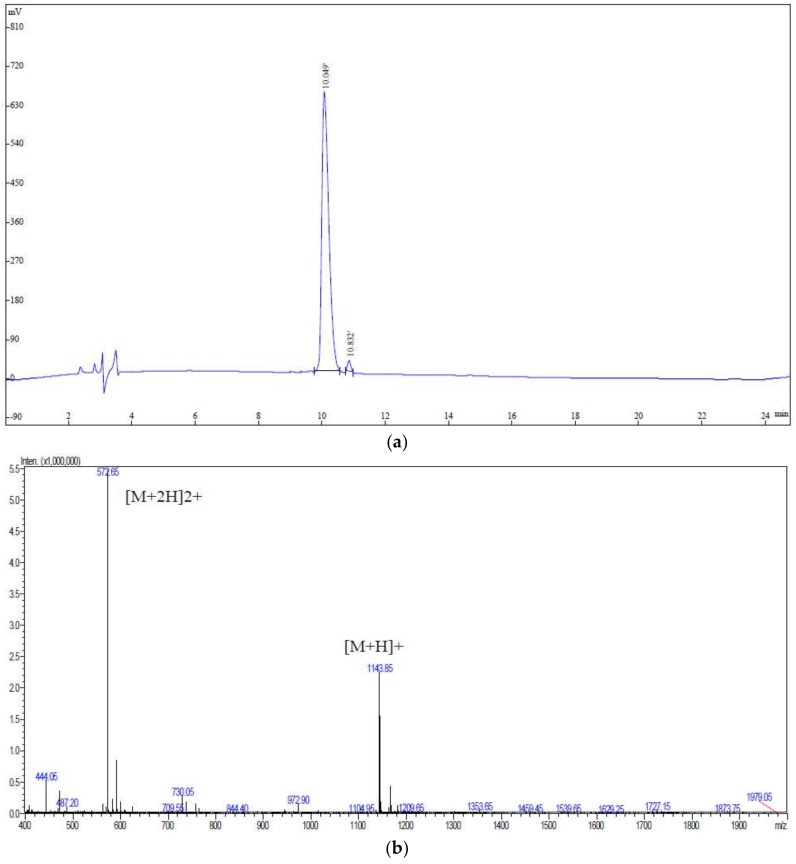
Identification of EPNGLLLPQY by (**a**) HPLC and (**b**) MS.

In the present study, in order to identify the peptides in the walnut hydrolysates, UPLC-Q-TOF-MS/MS combined with MASCOT searching was used to analyze the fragment spectra [24]. As shown in Table 1, in total, 6 kinds of proteins containing 33 unique peptides were identified from walnut protein hydrolysates. Many kinds of peptides were identified from walnut seed storage protein (Q2TPW5). Walnut seed storage protein consists of 507 amino acids, and it can probably release bioactive peptides by enzymatic digestion of pepsin and trypsin, and so on. Results of peptides along with the peptide sequences and molecular information are shown in Table 1. The Mascot searching program can only provide the information of the peptides with the number of amino acids more than six, otherwise the precision of the identification work may be impossible. Among the identified peptides derived from the hydrolysates of walnut seed storage proteins by trypsin, one peptide, EPNGLLLPQY (fragment residues 80–89), was predicted to be an ACE-inhibitory peptide according to the affinity analysis by molecular docking. Therefore, the EPNGLLLPQY was synthesized in order to clarify the mechanism of ACE-inhibitory activity. The HPLC and MS of the synthesized EPNGLLLPQY are shown as Figure 1. The peptide showed a retention time of 10.049 min in HPLC in Figure 1a with a high purity of 98.51%, which showed that it can be used for the following analysis. The molecular mass of the peptide was 1143.43 Da, which was determined by MS/MS as shown in Figure 1b.

In the present study, the peptide EPNGLLLPQY was identified from the hydrolysate of walnut protein digested by pepsin at 37 °C and pH 1.2 for 3 h. Moreover, there were several reports related to the peptides from walnut protein hydrolysates with ACE-inhibitory activity. Several ACE-inhibitory peptides prepared from walnut proteins, LPGRPPIKPWPL and WPERPPQIP, were identified and purified by column chromatography methods from the hydrolysate of walnut protein digested by neutral proteinase As1.398 at 45 °C for 5 h (pH 7.0) [12,20].

**Figure 2:**
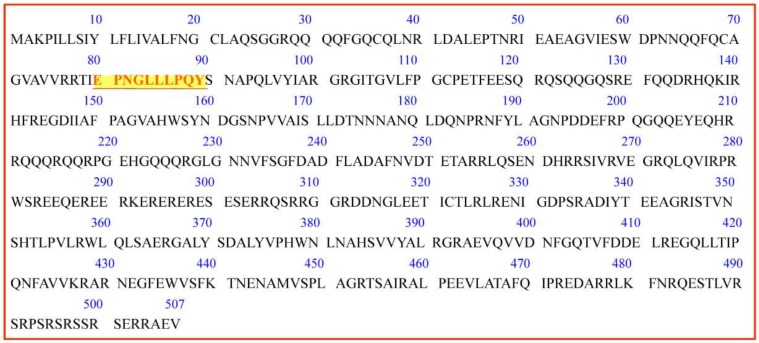
Location of EPNGLLLPQY in walnut seed storage protein (Q2TPW5).

To screen the ACEI peptide, several in silico methods (Peptide Ranker and molecular docking) were used in the present study. Firstly, as the molecular weights of the identified protein ranged from 702.366–2590.46 Da with the number of amino acids from 7–23 and the study paid more attention to the peptides with a lower molecular weight, the identified peptides with amino acids no more than 10 were selected for further study. Secondly, Peptide Ranker was adopted to analyze the possibility of the peptide to be bioactive. As listed in Table 2, the peptide EPNGLLLPQY acquired the highest scores of Peptide Ranker, so it has the highest likelihood to inhibit ACE activity. Thirdly, molecular docking was employed to verify the affinity of the peptides binding to ACE. It is not hard to see that the peptide EPNGLLLPQY also has a relatively high affinity with ACE, which was evaluated by comparing the scores of “-CDOCKER_Interaction_Energy”, an evaluation function of the affinity of the donor and ligand in molecular docking in Discovery Studio 2017. Therefore, the peptide EPNGLLLPQY was chose for further ACEI analysis. The molecular mass of the peptide was 1142.60 Da, which was determined by MS/MS. Moreover, the location of EPNGLLLPQY (marked in red letter) in walnut seed storage proteins (Q2TPW5) from the PDB database is shown in Figure 2.

### 2.2. ACE-Inhibitory Activity Determination

**Figure 3:**
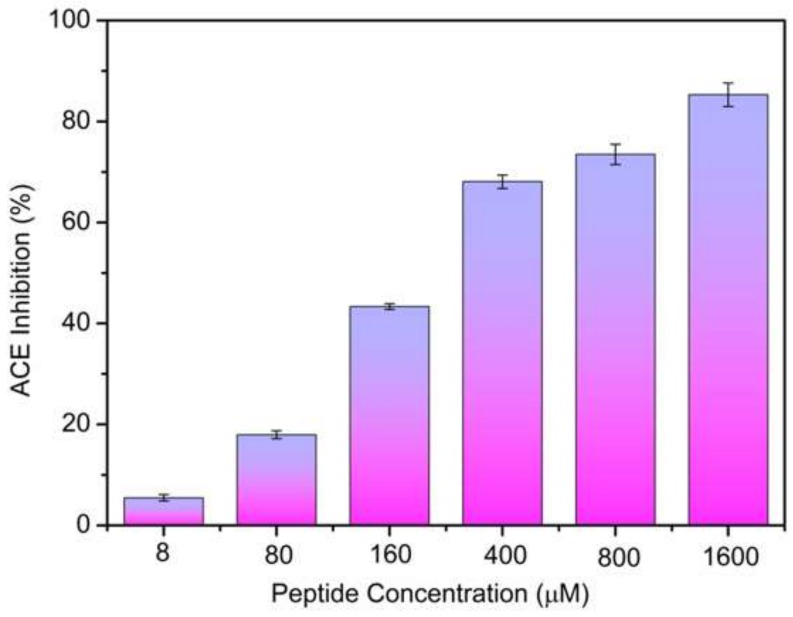
ACE-inhibitory activity of EPNGLLLPQY.

In the present study, the ACE-inhibitory activity of the peptide, EPNGLLLPQY, released by pepsin digestion was evaluated, as shown in Figure 3. The ACE-inhibitory activity of the peptide at different concentrations (8–1600 µM) was determined, the inhibitory activity increased with the increase of the concentration in a dose-dependent pattern. The IC_50_ value was determined by the regression equation: Y = −(1.93948 × 10^−4^)X^2^ + 0.24469X + 3.48868 (*R*^2^ = 0.97179). The IC_50_ value of the peptide was 233.178 µM. There have been about 700 kinds of ACE-inhibitory peptides from many kinds of proteins reported till now, all of which were collected in the BIOPEP database http://www.uwm.edu.pl/biochemia/index.php/en/biopep [25], and the IC_50_ values of the peptides ranged from 0.01–10,000 µM. The peptide EPNGLLLPQY can be considered as a potential ACE inhibitor due to its high activity.

### 2.3. Inhibition Pattern of the ACE-Inhibitory Peptide

**Figure 4:**
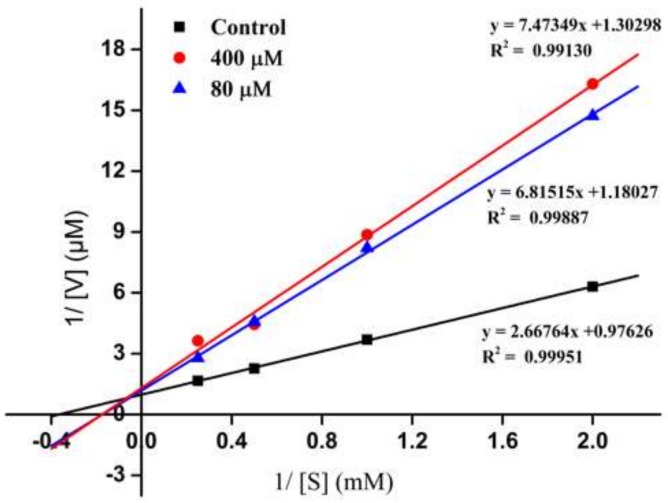
Line weaver-Burk plot of ACE inhibition by EPNGLLLPQY.

To further evaluate the active mechanism of the novel ACEI peptide, the inhibition pattern of the peptide, EPNGLLLPQY, against ACE was investigated by the Line weaver-Burk plot analysis method. As shown in Figure 4, three experimental groups (■ control; ▲ 80 μM; ● 400 μM) were tested, and 1/(S) and 1/(V) represent the reciprocal substrate concentration and velocity, respectively. The V_m_ values decreased as the peptide concentration increased, which confirmed that the existence of the peptide probably blocked the substrate from binding to ACE active sites. When the EPNGLLLPQY was added to the reaction system, the *K*_m_ values become higher than the control, which indicated that a relative higher content of substrate was essential for the ACE catalytic reaction. Figure 4 presents an ACEI peptide exhibiting a mixed-type of inhibition of ACE. Similar food-derived peptides EVSQGRP, VSRHFASYAN and SAAVGSP also have been identified from *Stichopus horrens* [26]. The mixed-type inhibition indicated that the peptide binds to the ACE at positions including both the active and non-active sites, which consequently reduces the catalytic activity of ACE. Since ACE converts the hormone angiotensin I to the active vasoconstrictor angiotensin II, binding on the active site will directly prevent the cleavage ability of ACE. Binding on the non-active sites probably prevents the interaction of the ACE and the substrate by the steric hindrance effect.

### 2.4. Activity Mechanism of EPNGLLLPQY

Molecular docking is a generally used method for the prediction of the interactions of the inhibitor as a donor and the enzyme as a receptor by calculating the affinity energies, indicating the binding sites and interactive bonds of the donor and receptor. In order to clarify the ACE inhibitive mechanism of EPNGLLLPQY, its molecular docking against ACE was performed by Discovery Studio 2017 R2 software. The values of “-CDOCKER_Interaction_Energy” (134.600 kcal mol^−1^) indicated the affinity between the EPNGLLLPQY and ACE, as shown in Table 2. Fu et al. [27] reported that the interactions of multiple hydrogen bonds between the inhibitor and ACE would promote the peptide-induced inhibition of ACE activity by the stabilization of the structure of the non-catalytic enzyme-peptide complex.

**Figure 5:**
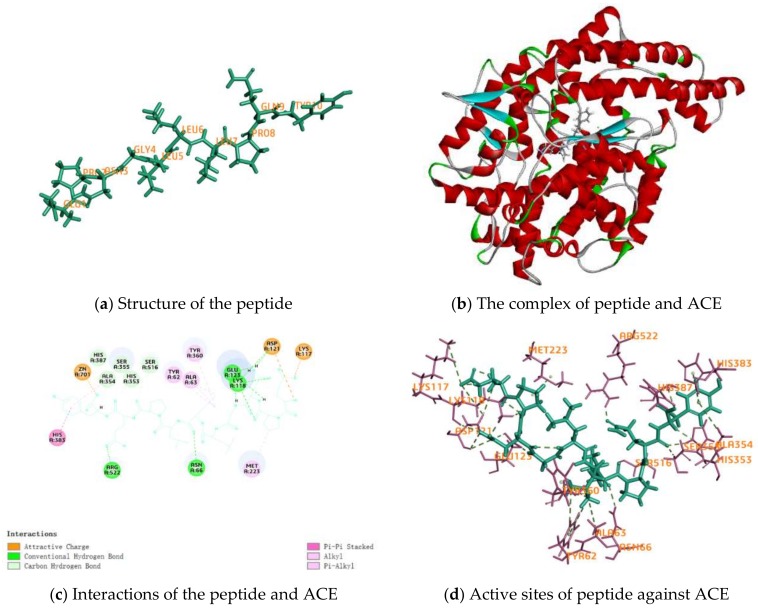

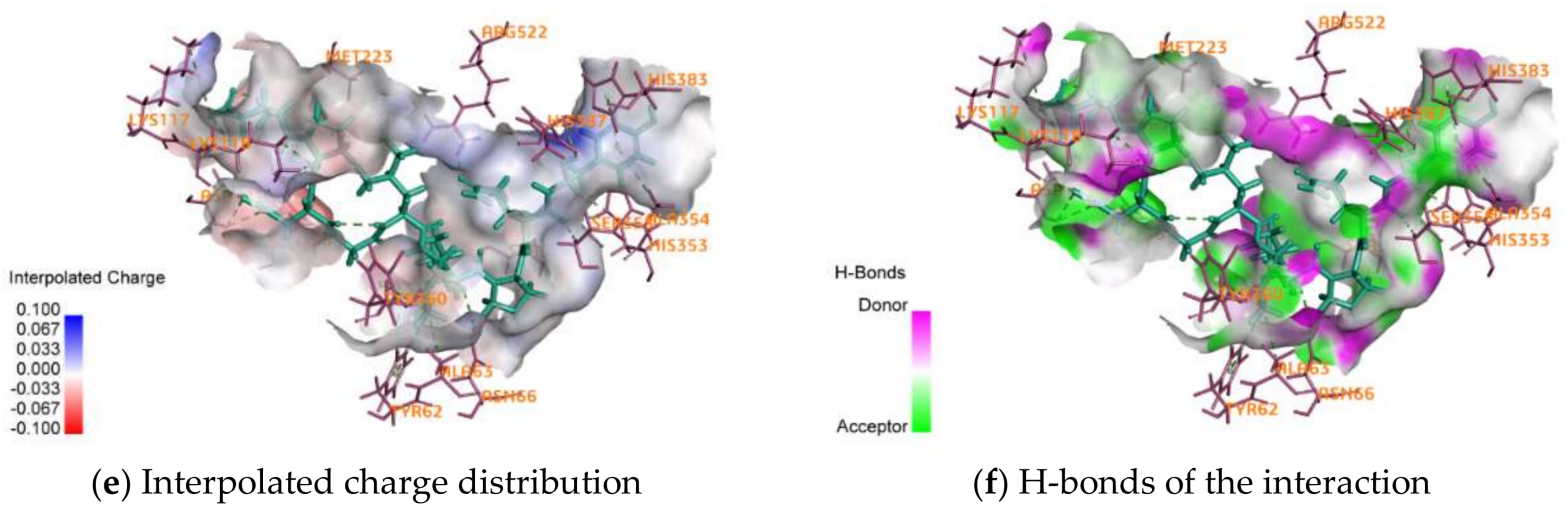
Molecular docking of EPNGLLLPQY against ACE (PDB: 1O8A).

The structure of the peptide and the complex of it and ACE are shown in Figure 5a,b, which indicated that the peptide was a linear peptide, and its size was important for the inhibitory activity against ACE. There are three categories of interactions between EPNGLLLPQY as the ligand and ACE as the donor, electrostatic, hydrogen bond and hydrophobic interactions, and the majority of interactions were hydrogen bonds (Table 3). A total of 16 H-bonds has been found in the interactive site between the peptide EPNGLLLPQY and ACE, which is also shown in Figure 5c,d, including 10 amino acid residues in ACE, i.e., ASN66, LYS118, ASP121, GLU123, HIS353, ALA354, SER355, HIS387, SER516 and ARG522, as shown in Figure 5e. Among these residues, GLU123 and ARG522 also formed H-bonds with the ACE-inhibitory peptides (WG and PRY), as reported by Fu et al. [27]. SER516 was also identified in the C-domain complex of ACE-BPPb [28]. The large amount of ACE residues formed in ACE peptides by H-bonds could explain the greater interaction between ACE and peptides. Seven hydrophobic interactions have been found in the interactive site between the peptide and ACE, including six amino acid residues in ACE, i.e., TYR62, ALA63, MET223, ALA354, TYR360 and HIS383. Moreover, electrostatic interactions at the site of LYS117, ASP121 and ZN701 in ACE-EPNGLLLPQY also contributed to stabilization, as shown in Figure 5f. These results suggested that the intensive interactions of EPNGLLLPQY with ACE may contribute to its strong inhibitory activity.

## 3. Materials and Methods

### 3.1. Materials and Chemicals

Defatted walnut meals were purchased from Pretty Woman Changbai (Dalian, Liaoning, China). Pepsin (EC3.4.23.1, 2500 units/mg protein) was purchased from Novozymes (Beijing, China). ACE, hippuryl-l-histidyl-l-leucine (HHL), acetonitrile and trifluoroacetic acid (HPLC) were supplied by Sigma Aldrich Co. (St. Louis, MO, USA). Formic acid (FA) (HPLC) was supplied by Fluka (Buchs, Germany).

### 3.2. Enzymatic Hydrolysis of Walnut Proteins

Defatted walnut meals were soaked in water and smashed, the mixture was stirred in a water bath of 55 °C for 1 h after adjusting the pH to 8.5 with 1 mol/L NaOH. Then, it was centrifuged at 3000× *g* for 10 min, and the supernatant was centrifuged at 4000× *g* for 20 min after adjusting the pH to 4.5 with 1 mol/L HCl. The sediment was washed by 95% ethanol to remove the fat, and the crude proteins were prepared by freeze drying. Crude proteins were digested by pepsin at 37 °C for 3 h (pH 1.2) with an enzyme/substrate (E/S) ratio of 1/50. The concentration of walnut protein was 50 mg/mL, which was determined by the Lowry method with minor modifications with bovine serum albumin as the standard [20]. The hydrolysis course was finished by heating the samples in boiling water (10 min). The supernatant from the hydrolysate was collected by centrifugation at 8000× *g* at 4 °C for 10 min.

### 3.3. UPLC-Q-TOF-MS/MS Analysis

The peptides in walnut hydrolysates were analyzed by an ultra-high-performance liquid chromatography (UPLC) quadrupole time of flight (Q-TOF) system using a Microtof-II mass spectrometer (Bruker Daltonik GmbH, Bremen, Germany). Isolation of peptides was performed on a UPLC instrument (Dionex UltiMate 3000 RSLC System, Thermo Scientific, Waltham, MA, USA) equipped with a 150 mm × 3.0 mm C 18 column (Phenomenex Luna, Torrance, CA, USA) with a particle size of 3.0 μm. Acidified water (0.1% formic acid, *v*/*v*) and ACN (with 0.1% formic acid, *v*/*v*) were used as Mobile Phases A and B. Elution was performed with a gradient of 5–35% B at a flow rate of 0.25 mL min^−1^.

The MS spectra were obtained in the positive electrostatic ionization manner. The top 20 most intense ions were acquired based on auto-MS/MS after the fragmentation of protonated molecular ions [29]. The raw data of the peptide sequence was processed by Data Analysis 4.0 software (Bruker Daltonics, Bermen, Germany) and searched by MASCOT (http://www.expasy.ch/sprot/, Version 2.5.1.0, Matrix Science, London, UK).

### 3.4. Screening of the Potential ACEI Peptides

To screen peptides, in silico methods were adopted. Molecular docking was used to evaluate the affinity between the peptides and ACE. Peptide Ranker was used to analyze the possibility of the peptides to be bioactive by giving them scores from 0–1, and the higher the score, the higher the possibility.

### 3.5. Synthesis of Peptides

Peptide with potential ACE-inhibitory activity screened by molecular docking was chemically synthesized by the solid-phase method (Cellmano Biotechnology Co., LTD, Hefei, China). The purity of the peptide was determined by HPLC, and the sequence of the synthesized peptide was determined by HPLC-MS/MS.

### 3.6. Molecular Docking

Molecular docking between walnut peptides and ACE was performed using Discovery Studio 2017 software (NeoTrident Technology Ltd., Beijing, China). The crystal structure of ACE (PDB ID: 1O8A) was downloaded from the PDB database. Based on their amino acid sequence, the structure and the minimum energy forms of target peptides were automatically figured out before molecular docking [30]. The types of interactions between the docked receptor and the ligand were analyzed.

### 3.7. ACE-Inhibitory Activity Assay

ACE-inhibitory activity was determined by an HPLC method (e2695, Waters, Framingham, MA, USA). The reaction system contained 30 μL of HHL (2.5 mmol L^−1^), 20 μL of ACE (100 μM) in 100 mmol L^−1^ of sodium borate buffer at pH 8.3 and 10 μL of the sample. The reaction was performed at 37 °C for 60 min and terminated by adding 60 μL of HCl (1 mol L^−1^). The concentrations of HHL and HA were determined by HPLC equipped with a Waters C-18 column (150 × 4.6 mm, 5 μm). To analyze the components, isocratic elution was conducted for 25 min in 78% Mobile Phase A (0.05% TFA in H_2_O) and 22% Mobile Phase B (ACN) with a flow rate of 0.5 mL min^−1^. The ACE inhibition (%) was calculated using the following equation:

$$\text{ACE Inhibitory Activity}\left(\%\right)=\frac{{\Delta A}_{\mathrm{blank}}-{\Delta A}_{\mathrm{inhibitor}}}{{\Delta A}_{\mathrm{blank}}}\times 100$$

where ΔA_blank_ and ΔA_inhibitor_ represent the peak areas of HA in the blank (sodium borate buffer) and sample, respectively. IC_50_ was defined as the concentration of the peptide that inhibited 50 percent of the ACE activity.

### 3.8. Statistical Analysis

All data were gathered in independent triplicates and described as the mean values and standard deviations. Statistics was performed using the SPSS 17.0 software.

## 4. Conclusions

A novel ACE-inhibitory peptide, EPNGLLLPQY, is a peptide (fragment of residues 80–89) located in walnut seed storage proteins, which exhibited inhibitory activity with an IC_50_ value of 233.178 µM in a mixed-type inhibition manner. The potential ACE-inhibitory mechanism was mainly attributed to the hydrogen bonds, hydrophobic and electrostatic interactions. Moreover, the peptides from walnut protein may be applied as a functional component in the research and development of functional products.

## Figures and Tables

**Table 1:** Walnut hydrolysate peptides identified by UPLC-Q-TOF-MS/MS.

No.	Protein	*m*/*z* Meas.	*z*	Mr. Calc.	Score	Start	AA Sequence	End	Length
1	Histidine kinase OS	529.3077	2	1056.593	38.4	L	LQLSALADRA	A	10
2	Legumin OS	832.4633	1	831.4491	21.37	L	LTIPQNF	A	7
3	Legumin OS	570.2736	2	1138.525	27.31	L	SAERGALYSDA	L	11
4	Legumin OS	648.374	2	1294.725	33.74	A	IRALPEEVLANA	L	12
5	Legumin OS	710.6773	3	2127.997	24.14	A	VALMDTTNNANQLDQNPRN	F	19
6	Legumin OS	759.702	3	2275.065	28.2	A	VALMDTTNNANQLDQNPRNF	Y	20
7	Legumin OS	864.8346	3	2590.46	29.51	A	VVRRTIEPNGLLLPQYSNAPQLL	Y	23
8	Legumin OS	670.3027	3	2007.871	24.94	L	MDTTNNANQLDQNPRNF	Y	17
9	Multi-sensor signal transduction histidine kinase	529.3077	2	1056.593	38.4	P	IQLSAERLQ	F	9
10	Receptor agonist peptide	520.9299	3	1558.77	21.66	A	KTPGLPPMPQSDYL	K	14
11	Seed storage protein	452.7325	2	903.445	28.1	A	FQIPRED	A	7
12	Seed storage protein	352.193	2	702.366	29.19	L	SAERGAL	Y	7
13	Seed storage protein	520.3148	2	1038.607	33.34	A	IRALPEEVL	A	9
14	Seed storage protein	574.2939	2	1146.567	41.27	L	ATAFQIPRED	A	10
15	Seed storage protein	555.8338	2	1109.644	22.87	A	IRALPEEVLA	T	10
16	Seed storage protein	529.3063	2	1056.593	38.51	W	LQLSAERGAL	Y	10
17	Seed storage protein	614.2948	3	1839.846	21.95	L	LDTNNNANQLDQNPRN	F	16
18	Seed storage protein	582.9686	3	1745.873	36.22	F	KTNENAMVSPLAGRTSA	I	17
19	Seed storage protein	994.97	2	1986.914	26.31	L	LDTNNNANQLDQNPRNF	Y	17
20	Seed storage protein	644.6458	3	1930.903	20.57	P	GEHGQQQRGLGNNVFSGF	D	18
21	Seed storage protein	921.4422	2	1839.854	62.21	F	PAGVAHWSYNDGSNPVVA	I	18
22	Seed storage protein	719.0287	3	2153.046	27.39	A	ISLLDTNNNANQLDQNPRN	F	19
23	Seed storage protein	827.139	3	2477.375	29.43	A	VVRRTIEPNGLLLPQYSNAPQL	V	22
24	Seed storage protein	526.9371	3	1577.78	26.95	F	LADAFNVDTETARR	L	14
25	Seed storage protein	863.9137	2	1725.803	80.77	L	LDTNNNANQLDQNPR	N	15
26	Seed storage protein	572.33	2	1142.60	27	-	EPNGLLLPQY	S	10
27	Seed storage protein	681.0131	3	2039.003	47.55	A	ISLLDTNNNANQLDQNPR	N	18
28	Seed storage protein	711.8505	2	1421.679	26.29	F	LADAFNVDTETAR	R	13
29	Vicilin-like protein precursor	432.7356	2	863.4501	25.24	L	LRGIENY	R	7
30	Vicilin-like protein precursor	425.7273	2	849.4345	37.36	Y	LRVFSND	I	7
31	Vicilin-like protein precursor	482.2702	2	962.5185	28.32	L	RVFSNDIL	V	8
32	Vicilin-like protein precursor	538.8131	2	1075.603	35.78	Y	LRVFSNDIL	V	9
33	Vicilin-like protein precursor	500.2511	3	1497.721	23.9	M	ESYFVPTERQSR	R	12

Mr. Calc.: Relative molecular mass calculated in MS analysis.

**Table 2:** Peptides analysis by Peptide Ranker and molecular docking.

No.	Peptides	Length	-CDOCKER_Energy Interaction (kcal mol^−1^)	Peptide Ranker
1	IRALPEEVL	9	155.937	0.14
2	LQLSALADRA	10	144.081	0.23
3	ATAFQIPRED	10	136.401	0.18
4	EPNGLLLPQY	10	134.600	0.37
5	LQLSAERGAL	10	130.532	0.30
6	LRVFSND	7	127.556	0.16
7	LRVFSNDIL	9	126.776	0.30
8	IQLSAERLQ	9	123.061	0.12
9	IRALPEEVLA	10	121.918	0.14
10	LTIPQNF	7	108.518	0.34
11	FQIPRED	7	97.6096	0.27
12	LRGIENY	7	94.0396	0.12
13	RVFSNDIL	8	85.8807	0.31
14	SAERGAL	7	68.027	0.29

**Table 3:** Interaction bonds of walnut peptide against ACE.

Interactions	Distance	Category	Types
A:LYS117:NZ–EPNGLLLPQY:O15	4.63933	Electrostatic	Attractive Charge
A:ZN701:ZN–EPNGLLLPQY:O162	2.22675	Electrostatic	Attractive Charge
EPNGLLLPQY:N1–A:ASP121:OD2	4.00046	Electrostatic	Attractive Charge
A:ASN66:HD21–EPNGLLLPQY:O90	2.53299	Hydrogen Bond	Conventional Hydrogen Bond
A:LYS118:HZ1–EPNGLLLPQY:O40	1.79716	Hydrogen Bond	Conventional Hydrogen Bond
A:LYS118:HZ3–EPNGLLLPQY:O31	2.13479	Hydrogen Bond	Conventional Hydrogen Bond
A:ARG522:HH12–EPNGLLLPQY:O135	1.92374	Hydrogen Bond	Conventional Hydrogen Bond
EPNGLLLPQY:H33–A:GLU123:OE1	1.97968	Hydrogen Bond	Conventional Hydrogen Bond
EPNGLLLPQY:H42–A:LYS118:O	2.29783	Hydrogen Bond	Conventional Hydrogen Bond
EPNGLLLPQY:H42–A:ASP121:O	2.83625	Hydrogen Bond	Conventional Hydrogen Bond
EPNGLLLPQY:H43–A:ASP121:O	2.80029	Hydrogen Bond	Conventional Hydrogen Bond
EPNGLLLPQY:H43–A:GLU123:OE2	2.2265	Hydrogen Bond	Conventional Hydrogen Bond
EPNGLLLPQY:H47–A:GLU123:OE1	2.01368	Hydrogen Bond	Conventional Hydrogen Bond
A:LYS118:HE2–EPNGLLLPQY:O31	2.33128	Hydrogen Bond	Carbon Hydrogen Bond
A:HIS353:HD2–EPNGLLLPQY:O140	2.40483	Hydrogen Bond	Carbon Hydrogen Bond
A:SER355:HB1–EPNGLLLPQY:O140	2.37051	Hydrogen Bond	Carbon Hydrogen Bond
A:HIS387:HD2–EPNGLLLPQY:O162	2.74483	Hydrogen Bond	Carbon Hydrogen Bond
A:SER516:HB1–EPNGLLLPQY:O123	2.80361	Hydrogen Bond	Carbon Hydrogen Bond
EPNGLLLPQY:H144–A:ALA354:O	2.26174	Hydrogen Bond	Carbon Hydrogen Bond
A:HIS383–EPNGLLLPQY	4.51235	Hydrophobic	Pi-Pi Stacked
A:ALA63–EPNGLLLPQY:C81	4.15055	Hydrophobic	Alkyl
EPNGLLLPQY–A:MET223	4.05541	Hydrophobic	Alkyl
A:TYR62–EPNGLLLPQY:C81	5.26359	Hydrophobic	Pi-Alkyl
A:TYR62–EPNGLLLPQY:C85	5.3078	Hydrophobic	Pi-Alkyl
A:TYR360–EPNGLLLPQY:C81	5.23035	Hydrophobic	Pi-Alkyl
EPNGLLLPQY–A:ALA354	4.78628	Hydrophobic	Pi-Alkyl
